# Ubiquity of *Polynucleobacter necessarius* subspecies *asymbioticus* results from ecological diversification

**DOI:** 10.1111/j.1462-2920.2010.02396.x

**Published:** 2011-04

**Authors:** Jan Jezbera, Jitka Jezberová, Ulrike Brandt, Martin W Hahn

**Affiliations:** Institute for Limnology, Austrian Academy of SciencesMondseestrasse 9, A-5310 Mondsee, Austria

## Abstract

The subspecies *Polynucleobacter necessarius asymbioticus* (> 99% 16S rRNA similarity) has a cosmopolitan distribution and a ubiquitous occurrence in lentic freshwater habitats. We tested if the observed ubiquity of these free-living planktonic freshwater bacteria results from a euryoecious (generalist) adaptation of *P. n. asymbioticus* strains, or from ecological diversification within the subspecies. We developed a reverse line blot hybridization assay enabling the cultivation-independent detection of 13 groups within the subspecies in environmental samples. A set of 121 lentic freshwater habitats, spanning a broad variety of habitat types (e.g. pH levels ranging from 3.8 to 8.5) was investigated for the presence of these 13 *P. n. asymbioticus* groups. Statistical analyses of the reverse line blot hybridization detections revealed pronounced differences in habitat preferences of several of the groups. Their preferences differed regarding pH, conductivity, dissolved organic carbon and oxygen concentration of habitats. For some groups, differences in environmental preferences resulted even in complete niche separation between them. The revealed differences in habitat preferences suggest that the previously reported ubiquity of *P. n. asymbioticus* results from ecological diversification within the taxon and not from generalist adaptation of strains.

## Introduction

Ubiquitous and cosmopolitan distribution is assumed for many species of free-living microorganisms, and plays central roles in the current ‘Everything is Everywhere’ debate on biogeography and distribution patterns of microbes (Finlay, 2002; Whitfield, 2005; O'Malley, 2008). Colonization of habitats broadly differing in physicochemical properties (e.g. acidic and alkaline lakes) and colonization of habitats located in different climatic zones (e.g. arctic and tropical zones) by a species or a species-like taxon could be explained by a euryoecious (generalist) adaptation, i.e. from an adaptation characterized by wide environmental and habitat tolerance ranges. On the other hand, such a broad distribution could also be explained by ecological diversification within the taxon resulting in subgroups characterized by rather stenoecious (specialist) adaptations (with narrow ecological tolerance and restricted habitat ranges). Such ecological diversification is usually resulting in organisms (ecotypes) occupying partially separated ecological niches (Begon *et al*., 2006; Walk *et al*., 2009). Species of macroorganisms with wide geographic distributions usually consist of many ecotypes differently adapted to local environmental conditions or to certain habitat types (Odum *et al*., 2004). In this study we tested if the cosmopolitan distribution and ubiquity of a certain bacterial subspecies in stagnant freshwater systems resulted from a euryoecious adaptation or from ecological diversification within the subspecies.

The subspecies *Polynucleobacter necessarius asymbioticus* (Hahn *et al*., 2009) serves as a model for a bacterial species, or species-like taxon with global geographic distributions encompassing a broad range of habitat variability. This subspecies contains all free-living strains affiliated with the species *P. necessarius*, while the only other subspecies, *P. n. necessarius*, contains exclusively obligate endosymbionts of ciliates affiliated with the genus *Euplotes* (Vannini *et al*., 2007; Hahn *et al*., 2009). The species *P. necessarius* (also known as PnecC bacteria, Hahn, 2003; Jezberová*et al*., 2010), which currently harbours only these two subspecies, represents a narrow phylogenetic taxon characterized by a minimal 16S rRNA gene similarity of > 99%. Thus, this taxon is comparable to operational taxonomic units frequently established in current diversity investigations on microbial communities by applying 99% sequence similarity thresholds to ribosomal sequences (Curtis *et al*., 2006).

Members of the subspecies *P. n. asymbioticus* represent heterotrophic freshwater bacteria with a planktonic lifestyle. Cosmopolitan distribution (Hiorns *et al*., 1997; Hahn, 2003; Pearce *et al*., 2003; Alonso *et al*., 2009; Hahn *et al*., 2009) and ubiquity in stagnant freshwaterhabitats (Jezberová*et al*., 2010) is well documented for this subspecies. *P. n. asymbioticus* inhabits a very broad range of freshwater systems including alkaline (Hahn, 2003; Wu and Hahn, 2006; Salcher *et al*., 2008) and acidic lakes and ponds (Burkert *et al*., 2003; Hahn *et al*., 2005; Percent *et al*., 2008; Taipale *et al*., 2009), oligotrophic high mountain lakes (Wu *et al*., 2006; Jezberová*et al*., 2010), eutrophic shallow lakes (Wu and Hahn, 2006), rivers (Sekiguchi *et al*., 2002; Crump and Hobbie, 2005), ponds in raised bog systems (Jezberová*et al*., 2010) and shallow temporary puddles on forestry roads (Jezberová*et al*., 2010). Even colonization of permanently (monimolimnion of a meromictic lake) or temporarily (hypolimnion of a holomictic lake) anoxic zones of stagnant systems was observed (Salcher *et al*., 2008; Jezberová*et al*., 2010). Application of a specific fluorescence *in situ* hybridization (FISH) probe revealed relative abundances of *P. n. asymbioticus* in natural habitats in the range of < 1% to 67% of total bacterial numbers (Hahn *et al*., 2005; Wu and Hahn, 2006; Salcher *et al*., 2008; Alonso *et al*., 2009; Jezberová*et al*., 2010). Conversely, detection of free-living *Polynucleobacter* bacteria in macro-environments like soil or saline systems (saline inland waters and eusaline marine systems) was never reported (Zwart *et al*., 2002; Hahn, 2003; Wu *et al*., 2006).

We investigated if *P. n. asymbioticus* bacteria present in different types of freshwater habitats represented different groups, and if the distribution of groups across the habitats was random or if they differed in habitat preferences. Reverse line blot hybridization (RLBH, Zwart *et al*., 2003) adopted to detection of distinct *P. n. asymbioticus* groups was used for screening of 121 freshwater systems for the presence of these groups. For this purpose, a set of 13 RLBH probes specifically targeting ribosomal internal transcribed spacer (16S–23S ITS) sequences of different *P. n. asymbioticus* groups were developed. Screened habitats are located in Central Europe and represent a broad variety of freshwater systems differing in many limnological parameters like water chemistry, trophic status, habitat size and altitude (Jezberová*et al*., 2010).

The phylogenetic resolution (i.e. intraspecific resolution) of the RLBH method was higher than methods used in previous studies to assess differences in the environmental preferences of closely related bacteria (Gray *et al*., 2004; 2007; Schauer *et al*., 2005; Newton *et al*., 2007). Our aim was to confirm or disprove the ubiquity by diversification hypothesis for *Polynucleobacter* bacteria (Jezberová*et al*., 2010).

## Results

### Development and validation of RLBH probes

**Fig. 1:**
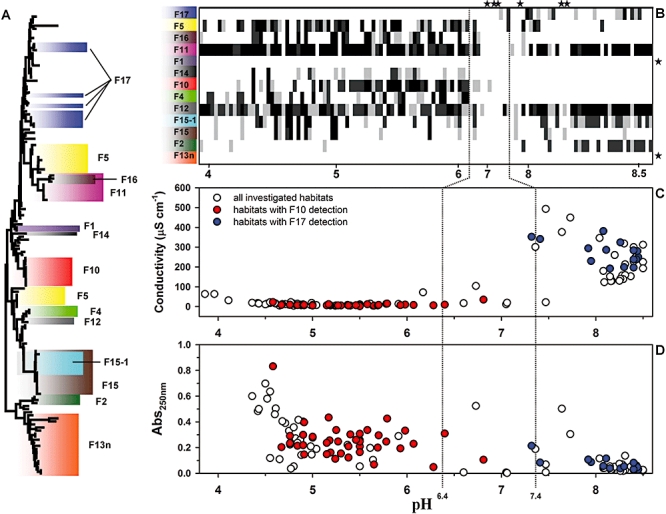
A. Neighbour-joining tree based on 16S–23S ITS sequences (about 500 bp, including two tRNA genes) of *P. n. asymbioticus* strains representing the culture collection (152 isolates) used for the development and testing of the RLBH probes. Lineages and groups targeted by the 13 probes are indicated by different colours. B. Detection by the RLBH probes across the 121 habitats, sorted by increasing pH (range 3.9–8.5). Note the non-linear scaling of the *x*-axis. Detection intensities are indicated by different gray scale colours; black, very strong; dark grey, average signal; light grey, weak signal; white, no detection signal. Habitats and probes without any detection are indicated by asterisks. C. Plot of conductivity values against pH values of all 121 habitats. Habitats with detections by probes F10 (red) and F17 (blue) are highlighted. D. Plot of absorption (250 nm) values representing a proxy for DOC and humic substance concentrations against pH values. Habitats with detections by probes F10 (red) and F17 (blue) are highlighted. Data of four habitats with absorption values > 1.0 are not shown (all four habitats were negative for F10 and F17).

Because of high similarity (> 99%) of 16S rRNA gene sequences of *P. n. asymbioticus* strains, it was impossible to use this phylogenetic marker to resolve the intra-subspecies diversity of *P. n. asymbioticus*. Therefore, the 16S–23S ITS was used as an alternative marker providing a much higher intraspecific resolution (Hahn *et al*., 2005). The development of a primer pair for specific amplification of *P. n. asymbioticus* ITS sequences, as well as the development of subgroup-specific probes was based on 152 sequences of *P. n. asymbioticus* strains, five sequences of *P. n. necessarius* strains (endosymbionts) and 34 sequences of *Polynucleobacter* strains affiliated with other species. The 152 *P. n. asymbioticus* strains shared ITS sequences with similarities in the range of 94–100%. Sequence similarities limited the possibility to develop a panel of group-specific probes covering all of the available 152 *P. n. asymbioticus* sequences; however, a set of 13 group-specific probes passed empirical validation (Table 1). This panel of probes covers 57% of the 152 strains (Fig. 1A). Most of these probes target groups (minimal intragroup ITS sequence similarities 98.4–100%) consisting of 2–17 strains while one probe targets only a single strain of the culture collection. More than half of the 13 groups could not be discriminated from other groups on the level of 16S rRNA sequences (Table 2). Of those 12 groups representing more than one strain, nine and seven appeared in phylogenetic trees of ITS sequences and partial glutamine synthetase gene sequences (glnA), respectively, as monophyletic groups supported by bootstrap values > 65% (Table 2, Fig. S1). The two broadest monophyletic groups in the ITS tree (F11 and F13n, Table 2) appear in the glnA tree split in two separated groups. Group F15-1 appears in the ITS tree as a monophyletic group nested in group F15; however, F15-1 strains differ from the remaining F15 strains only in two nucleotide substitutions in their ITS sequences.Empirical testing confirmed that the primer pair developed for amplification of ITS sequences of *P. necessarius* was specific for this species.

**Table 1:** Group-specific probes used in the RLBH assays, their 5′ to 3′ sequences, and melting temperatures (*T*_m_)

Probe name	Sequence	*T*_m_ (°C)
F1	CTAAGCGATTGTTAATTGTTTAGT	54.2
F2	TTATAAAGTTCTTAAATAGTACTTAAAGT	54.0
F4	ACCATCAGCAGCAGTGATA	54.5
F5	CCACCAATCAGCGTTGATA	54.5
F10	ACTAAGCGATCTAATGATTGTTTA	54.2
F11	MAGTGATATGGACTAWGTGG	54.2
F12	CCACCAATCAGCAGTGATA	54.5
F13n	CTAAGACCATATCACTACTGA	54.5
F14	ACTAAGCGATCTATTGATTGTTTA	54.2
F15	ACTAAGCAATTTAGCGATTGTTTA	54.2
F15-1	CCAAACTGTAAGTACTTATTAAAG	54.2
F16	GACCCACCAAATCAGAAGT	54.4
F17	CAAAACTAAGCGAAGACTTAATCGTTTAGTT	54.7

The probes target different regions of the 16S–23S ITS of *P. n.* ssp. *asymbioticus* strains.

**Table 2:** Number of strains in the strain collection targeted by the respective probes, accession numbers of reference sequences, minimal 16S–23S ITS sequence similarities of the groups defined by the respective probes, bootstrap values obtained for the respective groups in neighbour-joining and maximum likelihood trees, lack of resolution of 16S rRNA sequences (the respective group can not be discriminated from the listed groups by using 16S rRNA sequences), and detection frequency of the respective probes (% of investigated habitats)

Group	Number of strains targeted	Reference sequence (accession number)	Minimal ITS sequence similarity (%)	Phylogeny 16S–23S ITS^d^	Phylogeny glnA^d^	Lack of resolution of 16S rRNA sequences^a^	Detected in habitats (%)
PnecC	151	–	94.1			–	100^b^
F1	2	AM110078	100	Monophyletic	monophyletic	F5, F10, other groups^c^	0
F2	3	AJ550665	99.4	Monophyletic	monophyletic	–	17
F4	3	AJ879778	99.8	Monophyletic	monophyletic	F5, F12, F15, F15-1, other groups	24
F5	17	FN429717, AJ879801	98.6	Polyphyletic	paraphyletic	F1, F4, F10, F12, F15, F15-1, other groups	43
F10	10	AJ879783	99.8	Monophyletic	monophyletic	F1, F5, other groups	34
F11	9	AM110094, FN429704	98.4	Monophyletic	polyphyletic	–	79
F12	2	AM397064	99.6	Paraphyletic	monophyletic	F5, F15, F15-1, other groups	84
F13n	20	FN429677	98.4	Monophyletic	Polyphyletice	–	0
F14	1	FN429688	–	Not testable^d^	not testable^d^	–	16
F15	15	AM110090, AM110086	99.4	Monophyletic	monophyletic	F4, F5, F12, F15-1, other group	17
F15-1	7	AM110086	99.8	Monophyletic	paraphyletic	F4, F5, F12, F15, other group	37
F16	3	FN429704	99.6	Monophyletic	monophyletic	–	25
F17	10	AJ550654, FN429658	98.8	Paraphyletic	paraphyletic	other groups	12

a Strains affiliated to other groups possess identical or almost identical 16S rRNA sequences.

b Detected by FISH and PnecC-specific PCR (Jezberová*et al*., 2010).

c Not targeted by RLBH probes.

d Only one sequence available.

e Two of the 20 sequences form a separated group.

### Screening of habitats by RLBH

In total, 121 habitats were screened by RLBH for the presence of the 13 *P. n. asymbioticus* groups defined by the probes (Fig. 1B). These habitats were characterized in detail in Jezberová and colleagues (2010), who also showed the presence of *P. n. asymbioticus* in all 121 samples (results by FISH as well as *P. necessarius*-specific PCR). PCR amplification with the *P. necessarius*-specific primers resulted for all samples in amplicons of the expected length. The set of 13 probes detected at least one of the targeted groups in 115 habitats (95%). On average, 3.8 ± 1.8 groups were detected per habitat. In total, all but two groups could be detected in the 121 samples. Not detected was the F2 group, one of the smallest groups consisting of only two strains, and the other not detected group was F13n, the largest and most diverse group. The other probes detected their target groups in 15 (12%) to 102 (84%) habitats respectively (average 42.6 habitats, 35.2% of habitats).

Eight of the 13 groups targeted by RLBH probes contain strains, which were isolated from habitats included in the screened set of lakes and ponds (Table S1). In 88% of cases, the groups represented by the isolated strains could be detected by RLBH. For instance, several strains targeted by probe F17 were isolated from Lake Mondsee (Hahn, 2003) and Lake Hallstättersee. This probe was always positive for surface water samples from these two alkaline lakes (in total 10 samples). Isolation results and population composition in Loibersbacher pond 1 (Hahn *et al*., 2005) were also largely confirmed by the RLBH results. In all cases where isolation results could not be confirmed by RLBH, water samples used for isolation of strains and RLBH investigations have been taken at different dates, thus may represent distinct ecological situations.

### Distribution patterns of groups detected by RLBH probes

**Fig. 2:**
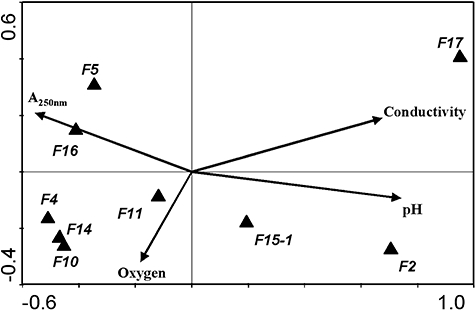
Canonical correspondence analysis of the environmental characteristics of the sampled habitats reveals correlations among single factors (arrows) and probe-defined groups of *P. n. asymbioticus* bacteria (triangles). This 2D model explains 38% of the observed variability. Only groups that showed significant correlations in this model are depicted. Oxygen, O_2_ concentration; *A*_250_, absorption at 250 nm.

Correlation analyses indicated that pooled data of pH and conductivity, as well as altitude and surface area best explained the observed distribution patterns of probe-targeted groups as shown by the redundancy analyses (RDAs) in Table 3. Conductivity and pH values of the investigated habitats are correlated (Fig. 1C). Altitude and surface area are also linked to pH and conductivity, because the acidic (low conductivity) habitats were mainly located at higher altitudes and possessed in all cases only small surface areas. Canonical correspondence analyses (CCAs) and RDAs revealed that *P. n. asymbioticus* groups most strongly differed in their preferences regarding pH, conductivity, DOC (absorption *A*_250_) and oxygen (Fig. 2 and Table 3), while water temperature did not significantly influence the composition of populations. Groups F4, F14 and F10 appeared as a tight cluster in the CCA analysis, while the paraphyletic group F17 possessed the most contrasting preferences compared with the cluster of those three groups. Groups F2 and F15-1 preferred habitats with higher pH and low oxygen concentration, and groups F16 and F5 preferred acidic habitats with higher DOC concentrations. Sorting of habitats by increasing pH (Fig. 1B) corroborates CCA results. Some groups (e.g. groups F4, F10, F14 and F16) showed clear preference for low pH habitats, other groups (i.e. mainly group F17 and to a minor extent group F2) displayed a preference to circum-neutral and alkaline habitats. Finally, some groups (i.e. F11 and F12) did not show clear preferences regarding pH of habitats.

**Table 3:** Results of the RDA calculated by CANOCO.

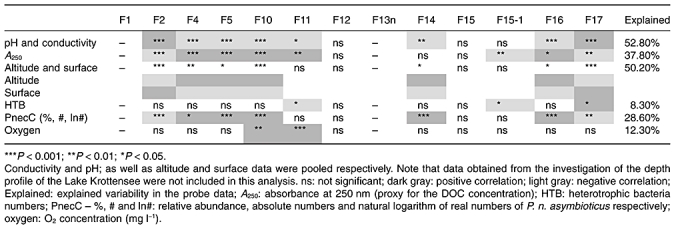

In general, habitats in the circum-neutral pH range (about pH 6.5–7.5) yielded low levels of *P. n. asymbioticus* detection with the probes, and the majority of the six samples lacking the detection of any group were characterized by circum-neutral pH values (Fig. 1B). The low detection frequency in the circum-neutral pH range can neither be explained by an underrepresentation of strains from such habitats in the culture collection nor by lower detection coverage of strains in this pH category by the panel of probes (Table S2). Importantly, detected maximal or minimal pH values of colonized systems fell for several of the groups in the circum-neutral pH range (i.e. groups defined by probes F4, F10, F14, F16 and F17), which indicates that those groups are either adapted to acidic or alkaline conditions.

**Fig. 3:**
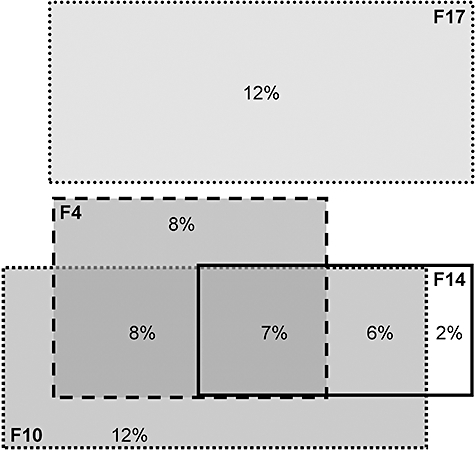
Venn diagram depicting separate and joined detection of the four groups F4, F10, F14 and F17 in the 121 habitats. The areas of the four rectangles, each representing one of the four groups, are proportional to the frequency of detection (% of habitats) respectively. The frequencies of sole, paired and triple occurrence are indicated by percentages of total number of detections. Of the habitats 45% lacked any detection with these four probes.

A sympatry-allopatry analysis revealed that groups F4, F10 and F14 were present in 43% of habitats and co-occurred in 21% (two or three of the groups) and 7% (all three groups) of the habitats, while group F17 did not co-occur at all with any of these three groups (Fig. 3). This suggests that differences in habitat preference (Figs 1B–D and 2) resulted in complete niche separation.

### Vertical distribution of RLBH detected groups in meromictic Lake Krottensee

Within-habitat differences in distribution of groups were investigated in meromictic (not completely mixing) Lake Krottensee. This lake is characterized by a permanently anoxic monimolimnion and a stable chemical stratification of the water column (Jezberová*et al*., 2010). Three groups of *P. n. asymbioticus* could be detected at the date of sampling of the vertical profile (May 2007; Fig. S2), while two additional groups could be detected in a sample taken at another date (June 2006; Table S1). Interestingly, two groups could only be detected in the oxygenated part of the water column; while the third group was also detected in samples from the anoxic zone.

## Discussion

Our goal was to reveal if the subspecies *P. n. asymbioticus* represents an ecologically coherent group, or if this taxon consists of subgroups differing in ecological adaptations and habitat preferences. Such knowledge is crucial for understanding the reported ubiquity of this subspecies in stagnant freshwater habitats (Jezberová*et al*., 2010) and is of general relevance for interpretation of ubiquity in other free-living microbes (Finlay, 2002). Several previous investigations demonstrated differences in ecological adaptation among closely related free-living bacteria. This was revealed either by relating spatial or temporal differences in natural occurrence of subgroups or genotypes to differences in environmental parameters (Gray *et al*., 2004; 2007; Schauer *et al*., 2005; Ahlgren and Rocap, 2006; Newton *et al*., 2007; Hunt *et al*., 2008) or by experimental demonstration of differences in adaptation (Jaspers and Overmann, 2004; Hahn and Pöckl, 2005; Sikorski and Nevo, 2007; Šimek *et al*., 2010). Most of these studies investigated differences in adaptation or habitat preferences in bacterial taxa, for which 16S rRNA similarity values of < 99% indicated affiliation to different species (Stackebrandt and Ebers, 2006). By contrast, the taxon investigated here represents a more closely related phylogenetic cluster characterized by a minimal intragroup 16S rRNA similarity of > 99%.

The definition of groups targeted by the RLBH probes was not based on multilocus sequence analysis of housekeeping genes; therefore, it could be that not each of these groups represents ecologically coherent groups (i.e. ecotype in Cohan, 2002). Indeed, the revealed polyphyly and paraphyly of some of the groups indicates that not all groups represent ecotypes. Lack of ecological coherence of groups could prevent the observation of ecological diversification using the RLBH approach. However, distributions revealed for most of the investigated *P. n. asymbioticus* groups showed both a non-random and a non-ubiquitous distribution pattern across the sampled habitats (Fig. 1B). This observation is further supported by statistical analysis (Fig. 2, Table 3), which suggested for the majority of groups significant differences in habitat preferences, which is best explained by differences in ecological adaptation. The three groups (F12, F11 and F15-1) with the broadest distribution across the wide range of freshwater habitats (Fig. 1B) appeared to be polyphyletic or paraphyletic groups in at least one of the two phylogenetic analyses (Table 2). Most likely, each of these groups does not represent an ecologically coherent group of strains with generalist adaptations, but ecologically heterogeneous groups consisting of two or more subgroups with distinct ecological adaptations. This is also indicated by the lack of detection of all three groups in the circum-neutral pH range, which argues for the presence of at least two subgroups, respectively, one better adapted to acidic and another better adapted to alkaline conditions. Ecophysiological characterizations and detailed phylogenetic investigations including multilocus sequence analysis will be required for testing the ecological and phylogenetic coherence of these broadly distributed groups (e.g. Walk *et al*., 2009).

An alternative explanation for the broad detection of some groups could be a lack of specificity of probes, i.e. match with *P. n. asymbioticus* subgroups not included in the culture collection and therefore not tested in empirical probe validation. In the case of group F15-1 such unspecificity could also explain the discrepancies in detection by probes F15 and F15-1. The group detected by the latter probe is nested in the broader group F15 but was more frequently detected as the umbrella group F15. Lack of specificity of probes could be indicated by discrepancies between pH range of habitats from which group members were isolated and pH range of habitats with RLBH detections (Fig. S3). The largest differences between the two ranges were observed for groups F15-1, F12, F11 and F2, which again indicate that detection patterns of the former three groups resulted from detection of ecologically heterogeneous groups respectively.

Two groups (F1, F13n) among the five groups lacking strains isolated from the investigated area could not be detected by RLBH in any of the sampled 121 habitats. All investigated habitats were located in Central Europe, while the 22 strains contained in these two groups were all isolated from habitats located outside of Europe. Therefore, lack of detection of these two groups could hint on absence of the groups in Europe because of restricted biogeographic distributions (Whitaker *et al*., 2003).

### Ubiquity of *Polynucleobacter* bacteria: generalist versus specialized adaptation

Our results do not support the hypothesis that ubiquity of *P. n. asymbioticus* in stagnant freshwater systems results from a generalist adaptation of these bacteria. In contrast, our findings support the ubiquity by diversification hypothesis (Jezberová*et al*., 2010). We could reveal clear habitat preferences and restricted ecological distributions for several of the investigated *P. n. asymbioticus* groups. The observed, relatively large, phenotypic diversity (e.g. substrate utilization patterns) among *P. n. asymbioticus* strains (Hahn *et al*., 2009) also suggests ecological diversification within this subspecies.

One may speculate whether this trend towards ecological specialization results from evolutionary constraints caused by small genome sizes of *P. n. asymbioticus* strains. Previous investigations, as well as genome sequencing of one strain, indicated that genome size of *P. n. asymbioticus* strains is in the range of 2.1–2.5 Mbp (Vannini *et al*., 2007; Hahn *et al*., 2009), which is significantly smaller compared with the majority of free-living bacteria sequenced so far (http://www.genomesonline.org). We speculate that such small genome sizes may not be sufficient for encoding numerous traits required for generalist adaptations.

### Factors controlling the genotypic composition of *P. n. asymbioticus* populations

The composition of *P. n. asymbioticus* populations in the investigated habitats can partially be explained by metapopulation theory (Levins, 1969; Hanski and Gilpin, 1997). According to this theory a metapopulation consists of a group of spatially separated populations of the same species that interact at some level. From a local metapopulation, habitats select for ecotypes with ecological adaptations to the environmental conditions present (ecotype sorting). Our statistical analyses indicated that pH adaptation and other unrevealed adaptations to water chemistry (indicated by conductivity and DOC concentration) were involved in ecotype sorting. This may result in the presence of a population consisting of ecotypes able to survive for longer periods of time in the habitat. The underlying mechanisms of this ecotype sorting by local environmental conditions is very similar to the concept of species sorting applied in metacommunity ecology (Leibold and Norberg, 2004; Van der Gucht *et al*., 2007). Seasonal changes in conditions, e.g. water temperature, may further modulate the seasonal genotypic composition of populations (Hunt *et al*., 2008). We did not observe a significant influence of temperature on the distributions of *P. n. asymbioticus* groups; however, our study design did not consider seasonal patterns and therefore, the temperature range covered by our sampling may have been too narrow to reveal significant influences. Additionally, the composition of local metapopulations could be influenced by regional dispersal limitations and potentially restricted biogeographic distributions of *P. n. asymbioticus* groups or ecotypes.

Results obtained in our investigation indicate that at least in the investigated subspecies of *P. necessarius*, ecological sorting by environmental factors takes place at the ecotype or subgroup level rather than the species level. This finding is in accordance with results of a couple of other investigations on freshwater, marine and soil bacteria (Jaspers and Overmann, 2004; Hahn and Pöckl, 2005; Sikorski and Nevo, 2007; Hunt *et al*., 2008; Walk *et al*., 2009; Šimek *et al*., 2010). If these findings represent a general evolutionary mechanism in a significant numbers of groups of bacteria, utilization of 16S rRNA genes for resolving community compositions may blur a large part of the ecologically relevant diversity of bacterial communities.

## Experimental procedures

### Habitats, sampling and basic limnological parameters

In total, 121 stagnant freshwater habitats (Table S1) were investigated for the presence of probe-defined *P. n. asymbioticus* groups. Detailed characterizations of these habitats have been presented elsewhere (Jezberová*et al*., 2010). Of the 137 habitats investigated previously (Jezberová*et al*., 2010) we included all but 13 habitats located in Sweden and three temporary puddles located in Austria, in the study presented here. The 121 habitats represent a large variety of lakes, ponds and permanent puddles, which differ in size and depth, trophic status, inorganic water chemistry and other limnological parameters. All habitats are located in Central Europe (Austria and the Czech Republic) and more than half of the habitats are located in a four-cornered area of 2353 km^2^ (Jezberová*et al*., 2010). All habitats were sampled at depths of about 0.5 m. In addition a depth profile of the meromictic Lake Krottensee was sampled (Jezberová*et al*., 2010). Measurements of physicochemical parameters and extraction of environmental DNA were performed as described previously (Jezberová*et al*., 2010).

### *P. n. asymbioticus* culture collection

A culture collection containing 152 isolates (Hahn, 2003; Hahn *et al*., 2005; M.W. Hahn, J. Jezbera, J. Jezberova and U. Koll, unpubl. data) was established by using either the filtration-acclimatization method (Hahn *et al*., 2004) or the dilution-acclimatization method (Hahn *et al*., 2005). Isolates were obtained from ecologically very contrasting habitats, including 24 (Table S1) of the 121 habitats investigated by RLBH. 16S–23S ITS sequences of the strains were obtained as described previously (Hahn *et al*., 2005). Partial sequences of the glutamine synthetase (glnA) gene were obtained by using primers glnA1212F and glnA1895R (5′-AGT WGC WCC WGT AGA TAC ATT CC-3′ and 5′-GTT GGG ATC TTT GCA TCT TCT TC-3′) (J. Jezberova, K. Simek and J. Jezbera, in preparation).

### *P. n. asymbioticus*-specific PCR amplification of ITS sequences

16S–23S ITS sequences of *P. n. asymbioticus* strains present in environmental samples were amplified by using the *P. n. asymbioticus*-specific forward primer PnecCf-4 (5′-CAC ACT TAT CGG TTG ACA ATA A-3′) and the biotinylated reverse primer PnecCr-5-BIO (5′-AAC GAG CAC CAT TGC TAG Y-3′), which specifically bind at the beginning and the end of the ITS of *P. necessarius* bacteria (Jezberová*et al*., 2010). The conditions of the PCR reaction were as follows: initial phase at 94°C (3 min), followed by 30 cycles of denaturation at 94°C (1 min), annealing at 61°C (1 min) and extension at 72°C (2 min). The final elongation at 72°C was run for 10 min. In case we obtained very low amount of PCR products from some habitats, we alternatively performed a nested PCR with PnecC441f (5′-GTC AGG GAA GAA ACA CCG-3′) and Poly23Sr primers (5′-GCT ACT TAG ATG TTT CAG TTC AC-3′), which bind specifically to the 16S and 23S rRNA genes of *P. n. asymbioticus* strains. The obtained PCRproducts were subjected to the above described PCR reaction for amplification of ITS sequences. The same PCR conditions were used for both steps of the nested PCR reaction. In all cases in which the one-step PCR yielded too low amounts of products, the nested PCR provided us with sufficient amount of labelled PCR products.

### RLBH probes development, testing and application

Reverse line blot hybridization was performed according to the protocol of Zwart and colleagues (2003) using 13 newly designed probes specifically targeting discriminative oligonucleotide sequences of the 16S–23S ITS of *P. n. asymbioticus* bacteria (Table 1). The probes were tested for specificity against all available isolates in our isolate collection. Used probes differed distinctly in probe signal intensities when applied to the same amounts of target PCR products (obtained from pure cultures of strains). For application of the method to environmental samples, probe concentrations were adjusted in order to compensate for differences in probe signal intensity (Zwart *et al*., 2003).

### Statistical and graphical analysis

Multivariable analyses were performed by using the CANOCO program (Ter Braak and Šmilauer, 1998). Detection data for probe-defined groups from each habitat were for statistical reasons analysed in nominal form [absent (0) or present (1)]. Unimodal undirect ordination such as CCA was selected for expressing the relationship between probe-defined groups and environmental variables (Fig. 2) based on the length of the gradient tested by detrended correspondence analysis, which was 4.3. Each environmental characteristic was also tested against all probe-defined groups by RDA, where environmental characteristics were imported as explained variables and genetic groups as explanatory variables (Table 3). Forward selection was used to choose significant explanatory variables in both CCA and RDA. Variables were included when *P* < 0.05 was estimated by a Monte Carlo permutation test with 999 unrestricted permutations. The results of the CCA analysis done in CANOCO were visualized by CanoDraw for Windows (Ter Braak and Šmilauer, 1998).

### Nucleotide sequences

16S–23S ITS sequences of *P. n. asymbioticus* strains used for probe design were deposited under the Accession Numbers AJ550654, AJ550657, AJ550664–AJ550666, AJ550670, AJ550671, AJ550673, AJ879778, AJ879783, AJ879801, AJ964893, AM110078–AM110083, AM110086–AM110091, AM110094–AM110097, AM110099–AM110102, AM110104, AM110105, AM110107, AM110109, AM110111–AM110113, AM397064–AM397066, FN429654, FN429655–FN429741, FN555148, FN556008, FN556009, FN825821–FN825839. GlnA sequences were deposited under the Accession Numbers FN823082–FN823233.

## Supporting information

**Fig. S1.** Comparison of phylogenetic trees based on complete 16S–23S ITS sequences (B) and partial glnA sequences (C). Two markers are located at almost opposite positions in the genome of *P. n.* ssp. *asymbioticus* strain QLW-P1DMWA-1 (A) (Accession Number NC_009379).

**Fig. S2.** Depth distribution of *P. n. asymbioticus* groups in meromictic Lake Krottensee (May 2007) as revealed by RLBH. Left graph, oxygen concentrations and total abundance of *P. n. asymbioticus* determined by a species-specific FISH probe (Jezberová et al., 2010). Note the non-linear scaling of the axis indicating the water depths. Right graph, detection of *P. n. asymbioticus* groups. All probes but probe F17 were applied to the samples. Besides the three presented probes, only application of probe F15-1 resulted in detections but the signals were very faint. Black – strong signal, dark grey – average signal, grey – weak signal, white – no signal. Note that sampling of the depths profile was performed on a different date than sampling of surface waters, which resulted in the detections presented in Table S2 and Fig. 1B.

**Fig. S3.** Comparison of pH ranges of investigated groups as indicated by RLBH (red) and pH range of habitats from which isolates were obtained (blue). Polyphyletic and paraphyletic groups are indicated by an asterisk.

**Table S1.** Number of *P. n. asymbioticus* strains isolated from habitats of different pH categories and number and percentage of them covered by the 13 employed RLBH probes. #, number.

**Table S2.** List of investigated habitats and detection of *Polynucleobacter necessarius* ssp. *asymbioticus* groups by RLBH probes. Detailed environmental data of these habitats were presented previously (Jezberová et al., 2010). Black rectangles indicate detection by a probe in at least one sample of the respective habitat, and red diamonds indicate isolation of at least one strain from the respective habitat. Note that strains were not always isolated from those water samples investigated by RLBH. PnecC (%), relative abundance of *P. n. asymbioticus* as % of total bacterial numbers (data from Jezberová et al., 2010).

## References

[b1] Ahlgren NA, Rocap G (2006). Culture isolation and culture-independent clone libraries reveal new marine *Synechococcus* ecotypes with distinctive light and N physiologies. Appl Environ Microbiol.

[b2] Alonso C, Zeder M, Piccini C, Conde D, Pernthaler J (2009). Ecophysiological differences of betaproteobacterial populations in two hydrochemically distinct compartments of a subtropical lagoon. Environ Microbiol.

[b3] Begon M, Townsend CR, Harper JL (2006). Ecology. From Individuals to Ecosystems.

[b4] Burkert U, Warnecke F, Babenzien D, Zwirnmann E, Pernthaler J (2003). Members of a readily enriched b-proteobacterial clade are common in surface waters of a humic lake. Appl Environ Microbiol.

[b5] Cohan FM (2002). What are bacterial species?. Annu Rev Microbiol.

[b6] Crump BC, Hobbie JE (2005). Synchrony and seasonality in bacterioplankton communities of two temperate rivers. Limnol Oceanogr.

[b7] Curtis TP, Head IM, Lunn M, Woodcock S, Schloss PD, Sloan WT (2006). What is the extent of prokaryotic diversity?. Philos Trans R Soc Lond B.

[b8] Finlay BJ (2002). Global dispersal of free-living microbial eukaryote species. Science.

[b9] Gray ND, Comaskey D, Miskin IP, Pickup RW, Suzuki K, Head IM (2004). Adaptation of sympatric *Achromatium* spp. to different redox conditions as a mechanism for coexistence of functionally similar sulphur bacteria. Environ Microbiol.

[b10] Gray ND, Brown A, Nelson DR, Pickup RW, Rowan AK, Head IM (2007). The biogeographical distribution of closely related freshwater sediment bacteria is determined by environmental selection. ISME J.

[b11] Hahn MW (2003). Isolation of strains belonging to the cosmopolitan *Polynucleobacter necessarius* cluster from freshwater habitats located in three climatic zones. Appl Environ Microbiol.

[b12] Hahn MW, Pöckl M (2005). Ecotypes of planktonic *Actinobacteria* with identical 16S rRNA genes adapted to thermal niches in temperate, subtropical, and tropical freshwater habitats. Appl Environ Microbiol.

[b13] Hahn MW, Stadler P, Wu QL, Pöckl M (2004). The filtration-acclimatization method for isolation of an important fraction of the not readily cultivable bacteria. J Microbiol Methods.

[b14] Hahn MW, Pöckl M, Wu QL (2005). Low intraspecific diversity in a *Polynucleobacter* subcluster population numerically dominating bacterioplankton of a freshwater pond. Appl Environ Microbiol.

[b15] Hahn MW, Lang E, Brandt U, Wu QL, Scheuerl T (2009). Emended description of the genus *Polynucleobacter* and the species *P necessarius* and proposal of two subspecies, *P necessarius* subspecies *necessarius* subsp nov and *P necessarius* subsp *asymbioticus* subsp nov. Int J Syst Evol Microbiol.

[b16] Hanski IA, Gilpin ME (1997). Metapopulation Biology: Ecology, Genetics and Evolution.

[b17] Hiorns WD, Methé EA, Nierzwickibauer SA, Zehr JP (1997). Bacterial diversity in Adirondack mountain lakes as revealed by 16S rRNA gene sequences. Appl Environ Microbiol.

[b18] Hunt DE, David LA, Gevers D, Preheim SP, Alm EJ, Polz MF (2008). Resource partitioning and sympatric differentiation among closely related bacterioplankton. Science.

[b19] Jaspers E, Overmann J (2004). The ecological significance of ‘microdiversity’: identical 16S rRNA gene sequences represent bacteria with highly divergent genomes and physiology. Appl Environ Microbiol.

[b20] Jezberová J, Jezbera J, Brandt U, Lindström ES, Langenheder S, Hahn MW (2010). Ubiquity of *Polynucleobacter necessarius* subsp *asymbioticus* in lentic freshwater habitats of a heterogenous 2000 km^2^ area. Environ Microbiol.

[b21] Leibold M, Norberg J (2004). Biodiversity in metacommunities: plankton as complex adaptive systems?. Limnol Oceanogr.

[b22] Levins R (1969). Some demographic and genetic consequences of environmental heterogeneity for biological control. Bull Entomol Soc Am.

[b23] Newton RJ, Jones SE, Helmus MR, McMahon KD (2007). Phylogenetic ecology of the freshwater *Actinobacteria* acI lineage. Appl Environ Microbiol.

[b24] O'Malley MA (2008). ‘Everything is everywhere: but the environment selects’: ubiquitous distribution and ecological determinism in microbial biogeography. Stud Hist Phil Biol Biomed Sci.

[b25] Odum E, Brewer R, Barrett GW (2004). Fundamentals of Ecology.

[b26] Pearce DA, van der Gast CJ, Lawley B, Ellis-Evans JC (2003). Bacterioplankton community diversity in a maritime Antarctic lake, determined by culture-dependent and culture-independent techniques. FEMS Microbiol Ecol.

[b27] Percent SF, Frischer ME, Vescio PA, Duffy EB, Milano V, McLellan M (2008). Bacterial community structure of acid-impacted lakes: what controls diversity?. Appl Environ Microbiol.

[b28] Salcher MM, Pernthaler J, Zeder M, Psenner R, Posch T (2008). Spatio-temporal niche separation of planktonic *Betaproteobacteria* in an oligo-mesotrophic lake. Environ Microbiol.

[b29] Schauer M, Kamenik C, Hahn MW (2005). Ecological differentiation within a cosmopolitan group of planktonic freshwater bacteria (SOL cluster, *Saprospiraceae, Bacteroidetes*). Appl Environ Microbiol.

[b30] Sekiguchi H, Watanabe M, Nakahara T, Xu BH, Uchiyama H (2002). Succession of bacterial community structure along the Changjiang River determined by denaturing gradient gel electrophoresis and clone library analysis. Appl Environ Microbiol.

[b31] Sikorski J, Nevo E (2007). Patterns of thermal adaptation of *Bacillus simplex* to the microclimatically contrasting slopes of ‘Evolution Canyons’ I and II, Israel. Environ Microbiol.

[b32] Šimek K, Kasalický V, Hornák K, Hahn MW, Weinbauer MG (2010). Assessing niche separation in coexisting *Limnohabitans* strains through interactions with a competitor, viruses, and a bacterivore. Appl Environ Microbiol.

[b33] Stackebrandt E, Ebers J (2006). Taxonomic parameters revisited: tarnished gold standards. Microbiol Today.

[b34] Taipale S, Jones RI, Tiirola M (2009). Vertical diversity of bacteria in an oxygen-stratified humic lake, evaluated using DNA and phospholipid analyses. Aquat Microb Ecol.

[b35] Ter Braak CJF, Šmilauer P (1998). CANOCO for Windows Version 4.02.

[b36] Van der Gucht K, Cottenie K, Muylaert K, Vloemans N, Cousin S, Declerck S (2007). The power of species sorting: local factors drive bacterial community composition over a wide range of spatial scale. Proc Natl Acad Sci USA.

[b37] Vannini C, Pöckl M, Petroni G, Wu QL, Lang E, Stackebrandt E (2007). Endosymbiosis *in statu nascendi*: close phylogenetic relationship between obligately endosymbiotic and obligately free-living *Polynucleobacter* strains (*Betaproteobacteria*). Environ Microbiol.

[b38] Walk ST, Alm EW, Gordon DM, Ram JL, Toranzos GA, Tiedje JM, Whittam TS (2009). Cryptic lineages of the genus *Escherichia*. Appl Environ Microbiol.

[b39] Whitaker RJ, Grogan DW, Taylor JW (2003). Geographic barriers isolate endemic populations of hyperthermophilic archaea. Science.

[b40] Whitfield J (2005). Biogeography: is everything everywhere?. Science.

[b41] Wu QL, Hahn MW (2006). Differences in structure and dynamics of *Polynucleobacter* communities in a temperate and a subtropical lake revealed at three phylogenetic levels. FEMS Microbiol Ecol.

[b42] Wu QL, Schauer M, Kamst-van Agterveld MP, Zwart G, Hahn MW (2006). Bacterioplankton community composition along a salinity gradient of sixteen high mountain lakes located on the Tibetan Plateau, China. Appl Environ Microbiol.

[b43] Zwart G, Crump BC, Kamst-van Agterveld MP, Hagen F, Han SK (2002). Typical freshwater bacteria: an analysis of available 16S rRNA gene sequences from plankton of lakes and rivers. Aquat Microb Ecol.

[b44] Zwart G, van Hannen EJ, Kamst-van Agterveld MP, van der Gucht K, Lindström ES, van Wichelen J (2003). Rapid screening for freshwater bacterial groups by using reverse line blot hybridization. Appl Environ Microbiol.

