# Supplementary material for: Ubiquity of Polynucleobacter necessarius subspecies asymbioticus results from ecological diversification
Source: Environ Microbiol. 2011 Apr;13(4):922–31. doi: 10.1111/j.1462-2920.2010.02396.x (PMC3087241; doi:10.1111/j.1462-2920.2010.02396.x)

Supplementary Material

Table S1. Number of *P.n. asymbioticus* strains isolated from habitats of different pH categories and number and percentage of them covered by the 13 employed RLBH probes. #, number.

pH	# of strains	% of strains	# of strains in category targeted by probes	% of strains in category targeted by probes
< 4	2	1	1	50
4 - 5	27	18	21	78
5 - 6	50	33	29	58
6 - 7	28	18	17	61
7 - 8	24	16	5	21
> 8	14	9	9	64
unknown pH	7	5	4	57

Table S2. List of investigated habitats and detection of *Polynucleobacter necessarius ssp. asymbioticus* groups by RLBH probes. Detailed environmental data of these habitats were presented previously (Jezberová et al., 2010). Black rectangles indicate detection by a probe in at least one sample of the respective habitat, and red diamonds indicate isolation of at least one strain from the respective habitat. Note that strains were not always isolated from those water samples investigated by RLBH. PnecC (%), relative abundance of *P. n. asymbioticus* as % of total bacterial numbers (data from Jezberová et al., 2010).

	Habitat	F1	F2	F4	F5	F10	F11	F12	F13n	F14	F15	F15-1	F16	F17	pH	PnecC (%)
**1**	Loiberbacher pond 1			■♦	■♦	■♦	■	■		■			■		4,7	51,4
**2**	Loiberbacher pond 2			■	■♦	■♦	■	■		■					4,8	21,7
**3**	Loiberbacher pond 3							■							4,8	15,7
**4**	Loiberbacher pond 4						■	■							4,7	14,1
**5**	Hintersee		■				■	■				■		■	8,5	1,5
**6**	Fuschlsee		■				■	■							8,5	6,4
**7**	Filblingsee							■						■	8,2	6,2
**8**	Eibensee		■					■						■	8,4	3,5
**9**	Irrsee		■					■							8,4	1,4
**10**	Mondsee (Scharfling)		■				■	■				■		■♦	8,3	0,2
**11**	Pond Seepromenade				■			■						■	7,4	7,5
**12**	Egelsee (Scharfling)				■		■	■						■	8,1	12,6
**13**	Krottensee		■				■	■				■		■	8,4	3.0
**14**	Wolfgangsee (Winkel)		■				■	■				■			8,4	0,4
**15**	Eislau							■							7,4	6,5
**16**	Schwarzensee		■				■	■				■			8,4	1,3
**17**	Mönichsee		■				■	■				■			8,3	1,1
**18**	Mittersee							■			■				8,2	0,5
**19**	Suisensee														8,2	0,6
**20**	Haleswiessee				■		■	■						■	8,0	15,5
**21**	Attersee (Unterach)		■				■	■				■		■	8,4	0,6
**22**	Egelsee (near Attersee)													■	7,9	3,4
**23**	Unterer Langbathsee						■	■				■			8,5	1,4
**24**	Oberer Langbathsee		■				■	■				■			8,5	0,7
**25**	Taferlklaussee														8,2	11,6
**26**	Traunsee (Altmünster)						■	■				■			8,3	1,1
**27**	Offensee		■				■	■				■		■	8,4	1,9
**28**	Nussensee						■	■			■	■		■	8,4	4,5
**29**	Altauseer See						■	■				■			8,2	0,5
**30**	Grundlsee						■	■				■			8,1	2.0
**31**	Toplitzsee						■	■				■			8,4	2,2
**32**	Sommersbergsee		■				■	■			■			■	8,1	2,7
**33**	Hallstättersee (Steeg)				♦		■	■				■		■♦	8,3	1,2
**34**	Vorderer Gosausee		■				■	■				■			8,3	6.0
**35**	Hinterer Gosausee		■				■	■				■			8,3	3,7
**36**	Gosau 3 (fishpond)						■								7,5	2,3
**37**	Vorderer Lahngangsee		■				■	■				■			8,3	1,5
**38**	Wildensee						■	■				■			8,3	0,2
**39**	Pond Sportland													■	7,3	9,1
**40**	Pond Rossmoss														6,7	40,1
**41**	Elmsee						■	■			■	■			8,1	2,7
**42**	Lacke near Elmsee				■	■		■							5.0	16,2
**43**	Post1			■	■	■	■	■							6,1	13,6
**44**	Post2			■	■	■	■	■		■					4,9	35,6
**45**	Post3			■			■	■							5,5	4,3
**46**	Post4			■♦	■	■	■	■		■					6.0	8,3
**47**	Illinger 1		■				■	■							4,6	33,8
**48**	Illinger 2				■			■							4,9	26,4
**49**	Egelsee I near Mattsee				♦										7,6	0,7
**50**	Egelsee II near Mattsee		■		■♦		■	■					■		7,7	0,2
**51**	Wengen Moor 1				■		■♦	■							4.0	0,5
**52**	Wengen Moor 2		■				■	■					■		3,9	> 0 §
**53**	Puddle near Wengen Moor						■	■							6,2	> 0 §
**54**	Wallersee				■			■							8,1	0,4
**55**	Pond 5 (garden pond)				■			■							8,3	8.0
**56**	Schwarze Lacke						■	■			■	■			4,4	11,1
**57**	Doppel-Lacke			■			■	■							4,5	0,7
**58**	Gerzinsel			■			■	■							4,4	17.0

Table S2 (continued)

**59**	Unterer Teich					■♦	■			■					5.5	6.7
**60**	Holzteich				■		■	■							4.5	26.0
**61**	Rechteckteich					■♦	■	■		■			■		5.5	22.5
**62**	Grosster Teich					■♦	■♦	■		■		■	■♦		5.5	27.9
**63**	Birnenteich			■	■	■	■			■			■		5.4	22.1
**64**	Pisateich					■	■						■		6.4	16.8
**65**	Kleiner Inselteich			■	■	■	■			■			■		5.5	27.4
**66**	Grosser Inselteich				■	■	■	■		■			■		5,0	17.3
**67**	Seggenteich			■	■	■	■	■			■		■		5.3	9.3
**68**	Stegteich			■	■	■	■	■							5.5	15.1
**69**	Trög 1				■	■	■	■			■	■	■		6.3	12.3
**70**	Trög 2			■	■	■	■	■		■	■	■	■		5.9	19.1
**71**	Trög 3			■	■	■	■	■		■			■		5.5	22.2
**72**	Trög 4			■			■	■							4.9	4.8
**73**	Trög 5				♦	■♦	■	■							4.8	24.2
**74**	Trög 6					■♦	■	■		■			■		5.2	17.9
**75**	Trög 7					■♦	■	■			♦				5.7	10.1
**76**	Trög 8		■	■	■♦	■♦	■♦	■							4.9	10.8
**77**	Letten1				■		■	■		■	■	■			4.9	20.5
**78**	Letten2			■	■	■	■	■							4.8	27.6
**79**	Letten3				■	■	■	■							4.9	20,0
**80**	Letten4					■									6.9	17,0
**81**	Reiter Alpe			■	■	■	■	■					■		5.3	20.5
**82**	Schwaig1				■		■	■							4.4	67.1
**83**	Schwaig2						■	■							4.6	34.2
**84**	Adlerlacke				■♦		■	■			♦	■♦	■		4.8	42.8
**85**	SchFe1				■		■	■				■			5,0	57.2
**86**	Horl1			■			■						■		5.6	31.3
**87**	Horl2				■		■					■	■		4.7	35.2
**88**	Horl3			■	■							■	■		5.4	34.5
**89**	Horl4				■		■	■				■	■		4.7	46.9
**90**	Salz1					■	■	■					■		5.4	1.9
**91**	Salz2					■	■	■		■		■	■		5.2	21.2
**92**	Salz4			■		■	■	■					■		5.7	27.2
**93**	Grasteich				■	■		■							5.6	37.2
**94**	Triangle Lacke				■			■				■			5.9	20.1
**95**	Oko Lacke				■	■	■	■				■			5.8	41.1
**96**	Kleiner Brauner Lacke			■	■	■	■	■							4.6	48.6
**97**	Kreuzungs Lacke				■	■	■	■							5.8	12.1
**98**	Portler Horn Teich			■	■	■	■	■		■					5.7	38.2
**99**	U-Lacke				■	■	■	■							5.2	15.5
**100**	Hidden Lacke					■	■								5.2	8.9
**101**	Joint Lacke			■	■	■	■	■							4.9	13.2
**102**	Jitka's Lacke					■	■	■							5.2	25.1
**103**	Furkla Lacke			■		■	■	■							5.2	6.9
**104**	Seilbahn Lacke I				■	■	■	■		■					5.4	31,0
**105**	Seilbahn Lacke II					■	■	■							5.3	43.8
**106**	Oberer Klaffersee							■							6.6	0.5
**107**	Unterer Klaffersee							■			■♦	■♦			7.5	1.1
**108**	Greifensteinsee														7.1	4.8
**109**	Rauhenbergsee														7.1	2.5
**110**	Gerlos1			■	■		■	■			■♦	■♦	■		4.8	10.9
**111**	Gerlos2			■			■	■			■	■			4.8	17.5
**112**	Gerlos3			■	■		■	■			■	■			4.6	25.5
**113**	Gerlos4				■		■	■			■♦	■♦	■		4.9	16.4
**114**	Gerlos5						■	■			■♦	■	■		5,0	11.4
**115**	Gerlos6				■♦		■	■			■	■	■		4.8	23.2
**116**	Chalupska Slat				■		■♦	■							4.1	22.1
**117**	Plešné				■		■	■		■	■	■	■		5,0	6.8
**118**	Čertovo						■	■							4.6	0.7
**119**	Černé			■	■		■	■			■♦	■♦			4.8	2.8
**120**	Prášilské				■		■	■		■	■♦	■♦	■		4.8	33.2
**121**	Laka				■		■	■			■	■	■		5.6	22.8

§ PnecC bacteria (*P.n.* ssp. *asymbioticus*) could be detected by FISH but could not be reliably quantified due to low cell numbers

Fig. S1. Comparison of phylogenetic trees based on complete 16S-23S ITS sequences (B) and partial glnA sequences (C). Two markers are located at almost opposite positions in the genome of *P. n.* ssp. *asymbioticus* strain QLW-P1DMWA-1 (A) (accession number NC_009379).

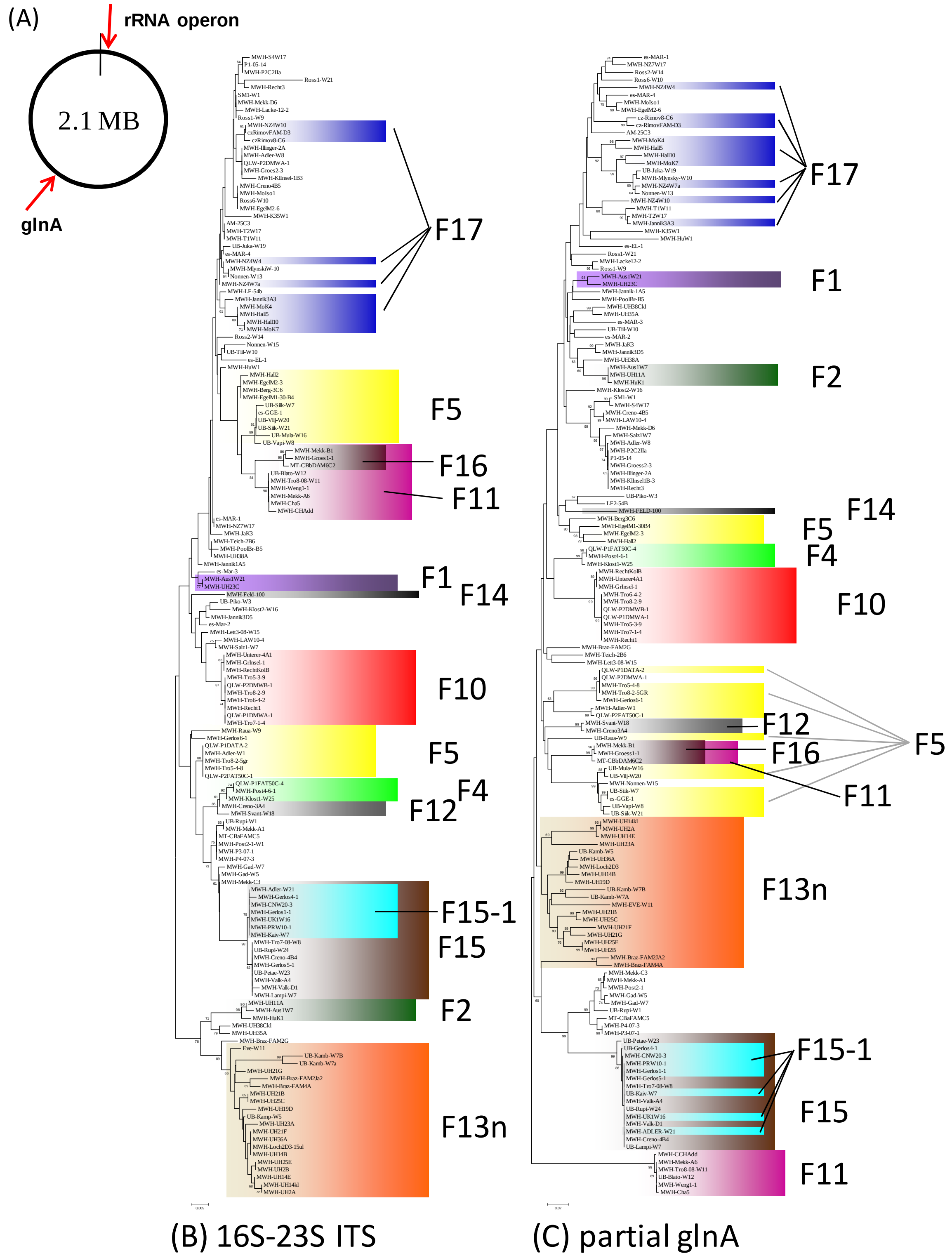

Fig. S2. Depth distribution of *P. n. asymbioticus* groups in meromictic Lake Krottensee (May 2007) as revealed by RLBH. Left graph, oxygen concentrations and total abundance of *P. n. asymbioticus* determined by a species-specific FISH probe (Jezberová et al., 2010). Note the non-linear scaling of the axis indicating the water depths. Right graph, detection of *P. n. asymbioticus* groups. All probes but probe F17 were applied to the samples. Besides the three presented probes, only application of probe F15-1 resulted in detections but the signals were very faint. Black – strong signal, dark grey – average signal, grey – weak signal, white – no signal. Note that sampling of the depths profile was performed on a different date than sampling of surface waters, which resulted in the detections presented in Table S1 and Fig. 1B.

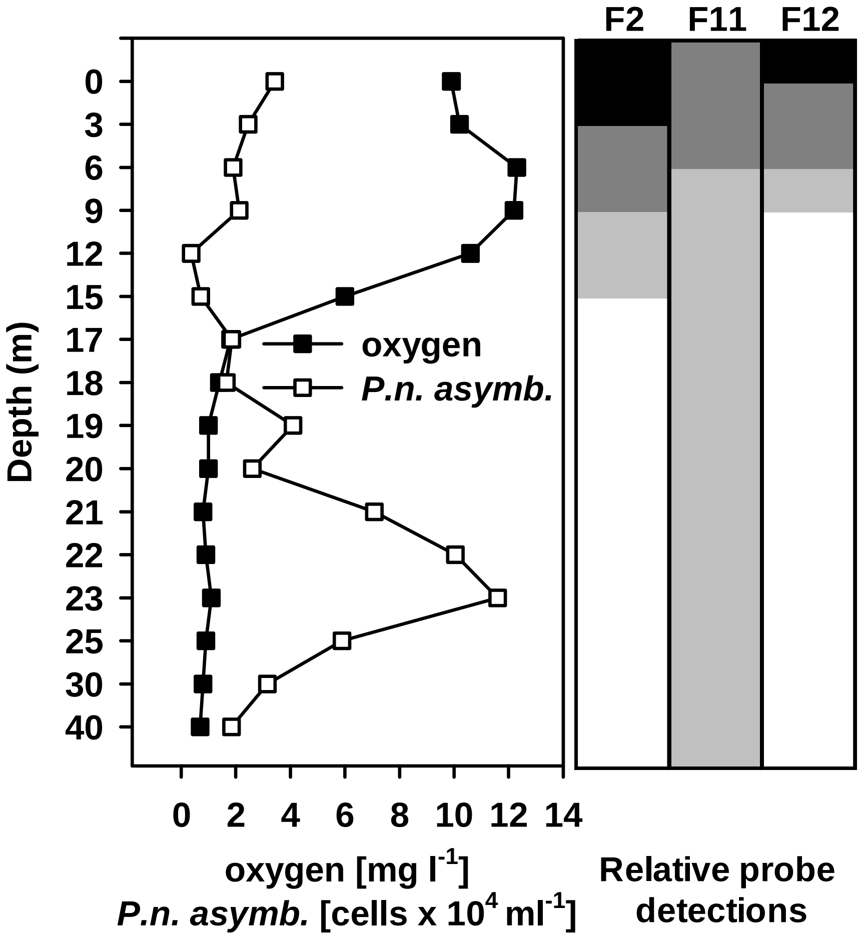

Fig. S3. Comparison of pH ranges of investigated groups as indicated by RLBH (red) and pH range of habitats from which isolates were obtained (blue). Polyphyletic and paraphyletic groups are indicated by an asterisk.

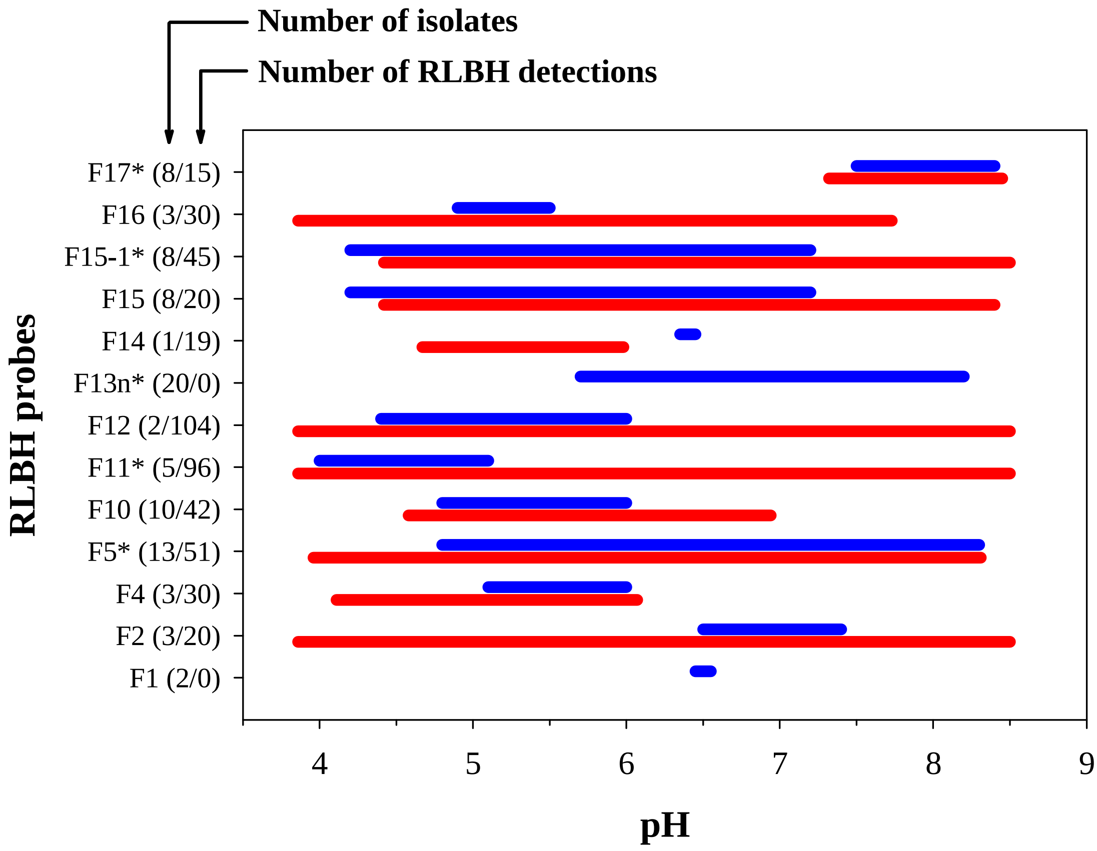

Supplement: Supplementary file 1 [file emi0013-0922-SD1.pdf]
